# Antidepressant Sales and the Risk for Alcohol-Related and Non-Alcohol-Related Suicide in Finland—An Individual-Level Population Study

**DOI:** 10.1371/journal.pone.0098405

**Published:** 2014-06-03

**Authors:** Heta Moustgaard, Kaisla Joutsenniemi, Mikko Myrskylä, Pekka Martikainen

**Affiliations:** 1 Population Research Unit, Department of Social Research, University of Helsinki, Finland; 2 Department of Mental Health and Substance Abuse Services, National Institute for Health and Welfare, Helsinki, Finland; 3 Department of Social Policy, London School of Economics and Political Science, London, United Kingdom; Chiba University Center for Forensic Mental Health, Japan

## Abstract

**Objectives:**

A marked decline in suicide rates has co-occurred with increased antidepressant sales in several countries but the causal connection between the trends remains debated. Most previous studies have focused on overall suicide rates and neglected differential effects in population subgroups. Our objective was to investigate whether increasing sales of non-tricyclic antidepressants have reduced alcohol- and non-alcohol-related suicide risk in different population subgroups.

**Methods:**

We followed a nationally representative sample of 950,158 Finnish adults in 1995–2007 for alcohol-related (n = 2,859) and non-alcohol-related (n = 8,632) suicides. We assessed suicide risk by gender and social group according to regional sales of non-tricyclic antidepressants, measured by sold doses per capita, prevalence of antidepressant users, and proportion of antidepressant users with doses reflecting minimally adequate treatment. Fixed-effects Poisson regression models controlled for regional differences and time trends that may influence suicide risk irrespective of antidepressant sales.

**Results:**

The number of sold antidepressant doses per capita and the prevalence of antidepressant users were unrelated to male suicide risk. However, one percentage point increase in the proportion of antidepressant users receiving minimally adequate treatment reduced non-alcohol-related male suicide risk by one percent (relative risk 0.987, 95% confidence interval 0.976–0.998). This beneficial effect only emerged among men with high education, high income, and employment, among men without a partner, and men not owning their home. Alcohol-related suicides and female suicides were unrelated to all measures of antidepressant sales.

**Conclusion:**

We found little evidence that increase in overall sales or in the prevalence of non-tricyclic antidepressant users would have caused the fall in suicide rates in Finland in 1995–2007. However, the rise in the proportion of antidepressant users receiving minimally adequate treatment, possibly due to enhanced treatment compliance, may have prevented non-alcohol-related suicides among men.

## Introduction

Suicide rates have declined markedly in recent decades in several countries [1]. This decline has, in many countries, co-occurred with an emergence and expansion in the use of new-generation antidepressants, namely selective serotonin reuptake inhibitors (SSRIs) and other non-tricyclic antidepressants [2], [3]. Since depression and other psychiatric disorders treated with antidepressants are major risk factors for suicide [4] it is plausible that more extensive mental health treatment has contributed to the decline in suicide rates. However, the causal connection remains debated [5], [6].

Suicide is a rare event and even large meta-analyses of randomized controlled trials have lacked statistical power to observe a significant effect of SSRIs on suicide risk, but have observed an *increase* in non-fatal suicide attempts during SSRI treatment [7], [8]. However, short follow-up times (8–10 weeks) of trials hinder the assessment of possible long time benefits of antidepressants [7]. A meta-analysis of large observational studies using individual-level data, with sufficient statistical power and longer follow-ups (2 months–7 years) reported a 40% decrease in odds of suicide among depressed adults and elderly using SSRIs [9]. A challenge with individual-level observational studies is the possibility of confounding by indication i.e. antidepressant use is not randomly allocated but is influenced by factors such as depression severity, suicidal ideation, and suicidal behavior, which are difficult to fully control for [9].

To overcome problems of confounding by indication many studies have used aggregate-level designs. Most aggregate-level studies have reported descriptive national trends or crude temporal correlations [1], although an increasing number of national [2], [10]–[15] and cross-national [16], [17] studies have used more sophisticated statistical methods, assessing regional level variation in antidepressant use and suicides, stratifying by age and gender, and controlling for regional changes in unemployment, divorce rates, and alcohol consumption. Aggregate-level studies have mostly found a significant beneficial effect of antidepressants on suicide rate in at least some age groups [1], [11]–[14], [16], [17]. However, they have rarely controlled for time trends and thus part of the observed association may be due to mere co-occurrence of two causally unrelated developments. It can be argued that time-controls are an over-adjustment [17] as they control for everything that is common across regions–including similar antidepressant sale trends. However, controlling for time is still the most stringent test for causality because it removes the confounding effects of all other co-occurring trends in observed and unobserved factors such as the national economy, alcohol consumption, and divorce rates that are common across regions, thus inferring effects only from the variation between regions in the changes in antidepressant sales. Studies controlling for time have yielded mixed results. A Norwegian study [2] only found an effect during times of low antidepressant sales, whereas a Swedish [10] and a Finnish study [15] found no effects in models where time was controlled for. The Finnish study [15] did report a significant effect from a model with multiple interactions between antidepressant use, region, time, age, and gender, but not for simpler models. Moreover the significant effect may need to be interpreted with caution as it would imply a much faster decline in suicide rates than has been observed in Finland.

One of the methodologically most convincing studies, with appropriate time-controls, exploited between-country variation in how new medication is adopted and diffused to predict the fall in suicide rates in 26 countries [16]. In countries where diffusion of new medication was early and quick, SSRIs tended to diffuse quickly as well, and this was associated with a more rapid fall in suicide rates, suggesting a 5% decrease in suicide rates per 1 pill per-capita-per-year increase in SSRI sales. A caveat with this study was that it studied total populations without differentiation of antidepressant exposure according to important population subgroups such as gender or socioeconomic group. Since antidepressant use is more common among women, whereas suicides are more common among men, combining genders in analyses is not without problems–an increased use of antidepressants among women may have little effect on suicides among men. The same applies for socioeconomic groups: in Finland suicide is more common among those with low social position [18], [19] while social group differences in antidepressant use are smaller or even reversed [20]. Antidepressants are likely to prevent suicide only in groups who are adequately treated. To the best of our knowledge, no previous study has assessed the association of antidepressants and suicide rate by social group.

Previous studies have also largely neglected different types of suicides. Alcohol-related suicides, i.e. suicides where alcohol intoxication is a contributory cause, are common among men and among individuals with low socioeconomic position [18], [19]. Alcohol-related suicides are also particularly common in depression, accounting for 35% of the excess suicides among depressed men and 10–20% among women [21]. It is unclear whether the expansion of antidepressant treatment has had beneficial effects on alcohol-related suicides.

### Current study

We use large Finnish register-based data to estimate the association between regional antidepressant sales and suicide risk. Our rationale is that where and when antidepressant sales are high, depressed individuals are more likely to be treated than where and when sales are low. If antidepressants reduce suicide risk, then the higher likelihood of being treated should be reflected in the lower likelihood of suicide. This assumption only holds if there are no regional and temporal differentials in factors leading to suicide, such as depression or alcohol consumption, and these evident differentials need to be controlled for. To do this we use a fixed-effects model that controls for all time invariant characteristics of regions as well as all national-level year-specific characteristics that may influence suicide risk irrespective of regional antidepressant sales. We add to the current literature in three ways. We (1) study non-alcohol-related and alcohol-related suicides separately; (2) assess the effects of antidepressant sales according to gender, socioeconomic position, employment status, and living arrangements in order to establish whether increased antidepressants sales have had similar effects across population subgroups; (3) use various measures for regional antidepressant sales–sold doses per capita, prevalence of antidepressant users, and the proportion of antidepressant users with doses reflecting minimally adequate treatment–in order to assess which dimensions of increased sales could explain the fall in suicide rates.

## Methods and Materials

### Ethics Statement

The study has been approved by Statistics Finland Board of Statistical Ethics (permit TK-53-1519-09). Informed consent from participants was not obtained, since the data were collected for routine administrative registration purposes and were anonymized prior to analysis.

### Data

Our data were based on individual-level registers on population, mortality, and health care covering all permanent residents of Finland. We obtained an 11% random sample of individuals aged 20 years or older at the end of any year 1994–2007, plus an 80% oversampling of individuals who died during this period (altogether 950,158 persons, 8,353,112 person-years, and 11,491 suicides). All analyses were weighted according to the differential sampling probabilities. The sample was drawn from population registers at Statistics Finland and included annual sociodemographic information and cause-specific mortality follow-up until the end of 2007. The sample was linked with individual-level information on all purchases of antidepressants in 1995–2007 from the registers of the Social Insurance Institution. Statistics Finland used personal identification numbers to combine data from different registers.

### Variables

#### Suicide

Suicides were classified according to the International Classification of Diseases (ICD) 9 and 10 (1995: ICD-9 codes E950–E959; 1996–2007: ICD-10 codes X60–X84, Y870). Suicides were divided into alcohol-related and non-alcohol-related according to whether the death certificate stated alcohol intoxication as one of the three contributory causes of death. We observed 2,859 alcohol-related and 8,632 non-alcohol-related suicides.

#### Antidepressant sales

Data on antidepressant sales came from the register of the Social Insurance Institution, which covers individual-level information on all prescription medication purchases gathered from all Finnish retail pharmacies, but excludes medication administered at hospitals and other institutional settings. In 1995–2007 the proportion of antidepressants sold by retail pharmacies increased from 89% to 96% [22], [23]. We used yearly information on purchases of non-tricyclic antidepressants (N06AB, N06AG, N06X, and N06AA22 in the Anatomical Therapeutic Chemical Classification System). Tricyclic antidepressants (other N06AA, currently around 7% of all sold antidepressant doses [24]) were excluded because little change in their sales has occurred in Finland in recent decades [24], and they are thus unlikely to have had major impact on declining suicide rates. Including tricyclic antidepressants in sensitivity analyses did not change the results.

Individual-level antidepressant use is not a suitable exposure in this setting because antidepressant users may be more likely to commit suicide due to their underlying mental health problems. We thus used regional sales of non-tricyclic antidepressants to indicate the antidepressant treatment possibilities individuals would have if they were depressed. We aggregated these from individual-level information on antidepressant purchases by region, year, and gender for the period 1995–2007. We used three measures for regional antidepressant sales:

1. The number of purchased defined daily doses (DDD) of non-tricyclic antidepressants per capita per year captures the amount of antidepressants sold overall. DDD is the assumed average daily dose of a given drug for its main indication, set by the World Health Organization, and is one of the most commonly used measure in previous aggregate-level studies on the antidepressant-suicide association [1].
2. The yearly regional prevalence of people with at least one antidepressant purchase reflects the proportion of the population who were actually using antidepressants. Among individuals with at least one purchase 76% had two or more purchases.
3. The proportion of individuals with a yearly minimum of 90 DDDs of antidepressant purchases among all individuals with at least one antidepressant purchase. This corresponds to a prescription of three months or more–the general length of the acute phase treatment of depression [25] –and is used as a proxy for the proportion of antidepressant users who received doses reflecting minimally adequate treatment. We also performed sensitivity analyses where we defined minimally adequate treatment as having a minimum of 180 DDDs of antidepressant purchases. This would correspond to acute and continuation phase of treatment.

Region of residence was updated annually and was measured by the so-called NUTS3 regions (n = 21) stipulated by the European Union, which roughly correspond to the Finnish hospital districts, with the exception of the Helsinki metropolitan area (cities of Helsinki, Vantaa, Espoo, and Kauniainen) separated from the surrounding region. In our sample the number of individuals per region varied from 5,210 to 182,199.

#### Social factors

In order to assess whether the rise in antidepressant sales has affected suicide risk similarly across social groups, we used annual individual-level measures of socioeconomic position, employment status, and living arrangements obtained from end-of-year registers of Statistics Finland. Education was based on highest achieved qualification and classified as more than nine years and nine years or less. Employment status was measured at the end of each year and included employed persons and others. Individual income tertiles were calculated from the combined population of men and women. The 2^nd^ and 3^rd^ tertiles were combined and compared with the lowest tertile. Homeowners were compared to those living in rented or other housing, and those living with a partner were compared to those without a partner. Antidepressant sales were not disaggregated by social factors.

### Statistical analyses

We used individual-level Poisson-models to examine how fluctuations in regional antidepressant sales were associated with changes in suicide risk in 1995–2007. We applied a region and time fixed-effects approach to control for all regional and temporal variation that may affect suicide risk irrespective of antidepressant sales [26]. We estimated three models. The age-adjusted Model 1 was a reference model that incorporated all variation between regions and years. This model is potentially biased by regional differences in mental health, since both suicide rates and antidepressant sales are likely to be high in regions with a high prevalence of depression, and the potential beneficial effect of antidepressants will be suppressed. In order to control for any time-invariant regional characteristics we added regional fixed effects in Model 2. In Model 3, we introduced national-level year fixed effects (as single-year dummy variables) to control for all variation in suicide risk common to all regions in a given year irrespective of their antidepressant sales.

Each year 1995–2007, all individuals present and aged 20+ at the last day of the previous year were followed up for death or end of year. The Poisson-model for binary data allowed us to assess suicide risk in relation to person-time rather than number of persons at risk [27], however, sensitivity analyses with logistic models yielded similar results. As the yearly individual records were not independent observations we calculated robust standard errors clustered at the individual level–clustering standard errors at the regional level yielded similar results. All analyses were performed separately for men and women adjusting for categorical age (20–34, 35–49, 50–64, 65–79, and 80+). STATA 11.2 was used in all analyses [28].

## Results

### National trends in suicide rates and antidepressant sales

**Figure 1:**
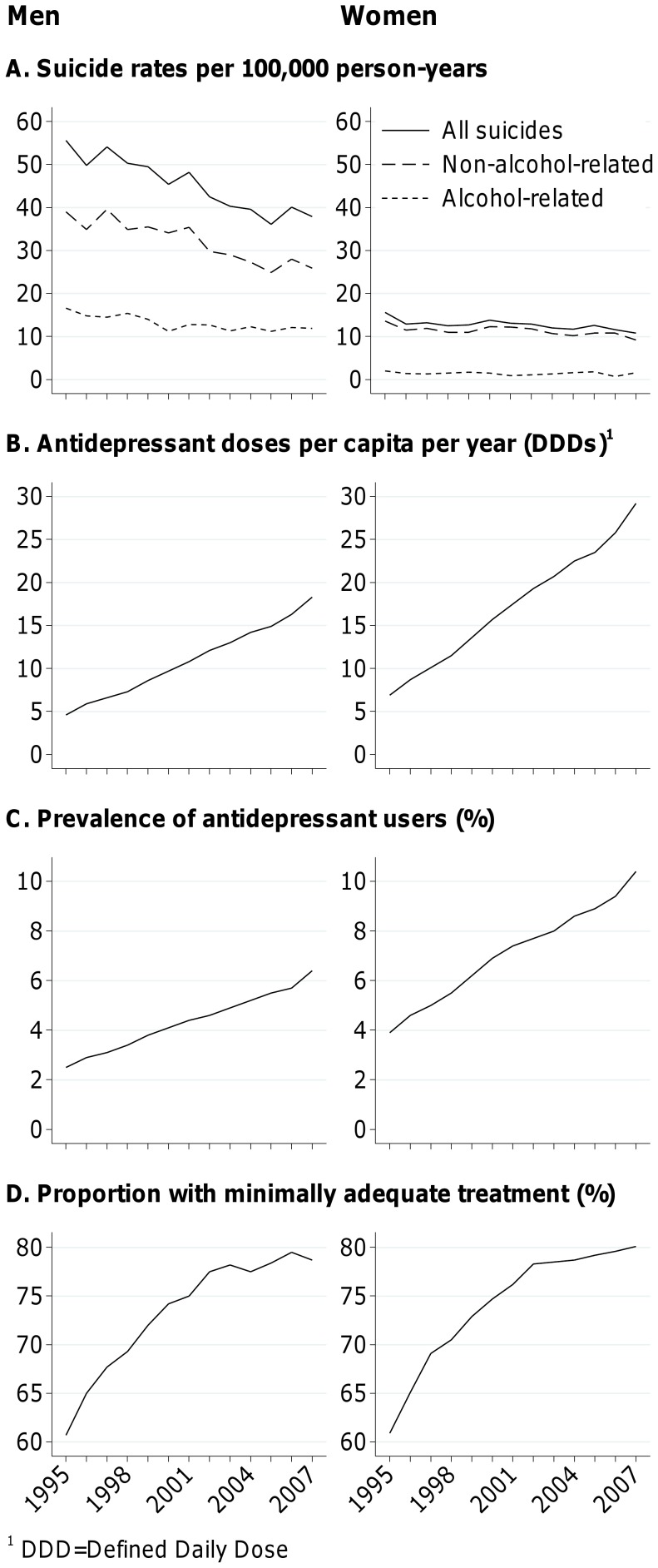
National trends in suicide rates and non-tricyclic antidepressant sales in Finland. Alcohol-related and non-alcohol-related suicide rates per 100,000 person-years (panel A), number of defined daily doses (DDDs) of antidepressants sold per capita per year (panel B), prevalence of antidepressant users (panel C), and proportion of individuals among all antidepressant users who have purchased at least 90 defined daily doses of antidepressants during a year (panel D) in 1995–2007 among Finnish men (left) and women (right) aged 20+.

Finland experienced a rapid decline in suicide rates in 1995–2007 (Figure 1). Most of this decline was accounted for by suicides where alcohol intoxication was not a contributory cause, whereas the decline in alcohol-related suicide rates was slower and stagnated altogether in the beginning of the 2000s. Around 30% of male suicides and 10–15% of female suicides were alcohol-related, the proportion slowly increasing among men. In the same period, non-tricyclic antidepressant sales increased steadily. The number of sold doses per capita went up from around 5–7 in 1995 to 18 among men and almost 30 among women in 2007. The 2007 sales correspond to an over two week daily use of antidepressants among men and a one-month daily use among women per capita per year. The prevalence of having at least one yearly antidepressant purchase more than doubled during the study period reaching 6% among men and 10% among women in 2007. The proportion of antidepressant users with doses reflecting minimally adequate treatment (i.e. at least 90 defined daily doses annually) increased from around 60% to 80% among both men and women.

### Regional suicide rates and antidepressant sales

To quantify the temporal connection between falling suicide rates and antidepressant sales we calculated regional correlations between age-adjusted suicide rates and antidepressant sales. Table 1 shows the average of these regional correlations weighted by population size. The correlations suggest that among men, regional antidepressant sales could explain at most 40% of the variation in non-alcohol-related suicide rates and around 10% in alcohol-related suicide rates. Among women antidepressant sales could only explain a maximum of 0–10% of the variation in suicide rates.

**Table 1:** Population-size weighted average of regional correlations between annually measured age-adjusted suicide rates and sales of non-tricyclic antidepressants in 1995–2007 (N = 273).

	All suicides		Alcohol-related		Non-alcohol-related	
	Men	Women	Men	Women	Men	Women
Sold doses per capita	−0.62	−0.29	−0.28	−0.05	−0.60	−0.31
Prevalence of users	−0.63	−0.28	−0.29	−0.05	−0.60	−0.30
% with minimally adequate doses	−0.61	−0.29	−0.32	−0.10	−0.57	−0.29

### Suicide risk by regional antidepressant sales

Table 2 shows the age-adjusted relative risk for suicide per one unit increase in regional antidepressant sales. Model 1 shows that one unit increase in the regional number of sold doses per capita was associated with a 3% decrease in the suicide risk of men and 1% decrease among women. One percentage point increase in the regional prevalence of antidepressant users was associated with a 9% decrease in male suicides and a 2% decrease in female suicides. Finally one percentage point increase in the proportion of antidepressant users receiving doses reflecting minimally adequate treatment decreased suicide risk by 2% among men and by 1% among women. These associations were largely similar in alcohol- and non-alcohol-related suicides.

**Table 2:** Age-adjusted relative risk (RR) of suicide per unit change in regional non-tricyclic antidepressant sales.

	Model 1		Model 2		Model 3	
	RR	95% CI^1^	RR	95% CI^1^	RR	95% CI^1^
Men (N = 463,807)						
All suicides (N = 8,814)						
Sold doses per capita	0.972	0.968–0.977	0.971	0.966–0.976	1.005	0.972–1.041
Prevalence of users	0.911	0.896–0.926	0.902	0.886–0.918	1.020	0.904–1.150
% with minimally adequate doses	0.982	0.978–0.985	0.982	0.979–0.985	0.993	0.984–1.003
Non-alcohol-related (N = 6,250)						
Sold doses per capita	0.973	0.967–0.978	0.969	0.963–0.975	1.000	0.959–1.042
Prevalence of users	0.913	0.895–0.931	0.895	0.876–0.914	1.034	0.895–1.195
% with minimally adequate doses	0.981	0.977–0.984	0.981	0.977–0.984	0.987	0.976–0.998
Alcohol-related (N = 2,564)						
Sold doses per capita	0.972	0.963–0.980	0.977	0.968–0.987	1.019	0.958–1.083
Prevalence of users	0.905	0.877–0.934	0.918	0.888–0.950	0.999	0.805–1.238
% with minimally adequate doses	0.983	0.978–0.989	0.985	0.979–0.991	1.006	0.989–1.023
Women (N = 486,351)						
All suicides (N = 2,677)						
Sold doses per capita	0.994	0.989–0.999	0.990	0.984–0.995	1.005	0.960–1.053
Prevalence of users	0.982	0.964–1.000	0.966	0.948–0.985	1.025	0.860–1.222
% with minimally adequate doses	0.990	0.984–0.996	0.990	0.984–0.995	1.000	0.976–1.023
Non-alcohol-related (N = 2,382)						
Sold doses per capita	0.994	0.989–1.000	0.990	0.984–0.996	0.997	0.949–1.048
Prevalence of users	0.983	0.965–1.002	0.966	0.947–0.986	1.021	0.846–1.231
% with minimally adequate doses	0.991	0.985–0.997	0.990	0.984–0.996	0.997	0.972–1.022
Alcohol-related (N = 295)						
Sold doses per capita	0.992	0.975–1.009	0.991	0.974–1.008	1.060	0.935–1.202
Prevalence of users	0.968	0.915–1.025	0.965	0.908–1.025	1.052	0.642–1.725
% with minimally adequate doses	0.985	0.968–1.002	0.986	0.969–1.004	1.025	0.956–1.098
Region fixed effects		no		yes		yes
Year fixed effects		no		no		yes

1 CI = confidence interval.

Model 1 is likely to be biased by regional differences in mental health and we thus controlled for all time-invariant regional characteristics by adding regional fixed effects in Model 2. As expected, the associations between antidepressant sales and suicide risk strengthened slightly. However in Model 3, when introducing the year fixed effects that control for all variation in suicide risk common to all regions irrespective of their antidepressant sales, only the regional proportion of antidepressant users receiving minimally adequate treatment was significantly associated with male risk for non-alcohol-related suicide, with one percentage point increase in proportion predicting 1% reduction in suicide risk. Controlling for a linear national time-trend or separate regional linear time-trends instead of year dummies, as well as assessing suicide risk with a one-year lag with respect to antidepressant sales, gave similar, yet non-significant, results (see Table S1). Including tricyclic medication in the antidepressant sales did not change the results shown in table 2. However, defining minimally adequate treatment as a minimum of 180 instead of 90 daily doses per year changed the results somewhat: the increase in the proportion of antidepressant users having at least 180 doses had no effect on suicide risk (Table S1).

### Variation according to social factors

We also assessed whether the association between antidepressant sales and suicide risk varied by social factors. As the overall effect was significant only for the proportion of antidepressant users receiving doses reflecting minimally adequate treatment and non-alcohol-related suicide among men, we present the social-group-specific analyses for this association only. Table 3 shows estimates from age-adjusted models including region and year fixed effects, stratified by social factors. The p-values for effect modification by social group come from interaction models including interactions of the given social factor with antidepressant sales, age, region, and year. Although according to the p-values, the effect of antidepressant sales was not significantly modified by any of the social factors, the significant decrease in suicide risk according to regional antidepressant sales was observed only in some groups. Higher regional proportion of antidepressant users with doses reflecting minimally adequate treatment reduced suicide risk among men not living in owned housing and not living with a partner, but not among homeowners and the partnered. There was also a borderline significant reduction in the suicide risk of men with more than 9 years of education, with higher income, and the employed.

**Table 3:** Age-adjusted relative risk (RR) for non-alcohol-related suicide per unit change in the proportion of antidepressant users who receive doses reflecting minimally adequate treatment by social factors among men (N = 463,807).

		% of person-years	RR	95% CI^1^	p^2^
Education, years	9+	64.1	0.984	0.968–1.000	0.550
	9 or less	35.9	0.991	0.975–1.007	
Employed	Yes	57.5	0.985	0.971–1.000	0.629
	No	42.5	0.991	0.973–1.009	
Individual income tertile	2nd or 3rd	77.1	0.985	0.971–1.000	0.565
	Lowest	22.9	0.992	0.974–1.010	
Home owner	Yes	69.0	0.993	0.973–1.014	0.478
	No	31.0	0.984	0.971–0.998	
Living with a partner	Yes	64.4	0.997	0.978–1.015	0.213
	No	35.6	0.982	0.968–0.996	
All		100	0.987	0.976–0.998	
Region fixed effects			yes		
Year fixed effects			yes		

1 CI = confidence interval.

2 p-value for significance of effect modification by social factor.

We further modelled associations of all measures of antidepressant sales with all measures of suicides for both genders in population sub-groups but found no other significant effects (data not shown). Among men a somewhat consistent pattern emerged with a beneficial effect of higher antidepressant sales among the employed, those with higher income, homeowners, and the partnered, as well as those with lower education, and a reversed effect among all others. Among women, all effects were weak, inconsistent and non-significant.

## Discussion

### Principal findings

Our aim was to elaborate the debated causal connection between the rapid increase in antidepressant sales and the co-occurring decline in suicide rates by applying a region and year fixed-effects model which controls for all time invariant characteristics of regions and all year-specific characteristics that may influence suicide risk irrespective of regional antidepressant sales. All in all, our results provide little support for the claim that increase in overall sales or in the prevalence of non-tricyclic antidepressant users would have caused the fall in suicide rates in Finland in 1995–2007. However, our results suggest a beneficial effect of increased antidepressant treatment among men—one percentage point increase in the regional proportion of antidepressant users with minimally adequate treatment (i.e. 90 DDDs or more annually) decreased the risk for non-alcohol-related male suicide by 1%. This implies that, given the observed increase in antidepressant sales, around half (63% with a 95% confidence interval 11%–105%) of the observed decline in non-alcohol-related male suicide rate could be explained by increased proportion of antidepressant users receiving minimally adequate treatment. The beneficial effect was observed among men with higher education, higher income, and the employed, and among those *not* owning their home and *not* living with a partner, whereas among other social groups there was no effect. Alcohol-related suicide and female suicide were unrelated to regional antidepressant sales.

### Potential explanations

Improved detection and adequacy of pharmacotherapy of major depression has been reported for Finland during 1989–2001, possibly due to an extensive suicide prevention program implemented nationally in 1992–1996 [29]. Our results suggest that the increasing proportion of antidepressant users receiving minimally adequate treatment had a beneficial effect, whereas mere per-capita sales and the prevalence of antidepressant users were unrelated to suicide risk. Short-term and non-psychiatric use of antidepressants has been found common in Finland and in other countries [30], [31]. If much of the rapidly expanding sales are accounted for by such short-term and non-psychiatric use then the effect on suicides is likely to be small. The increasing antidepressant sales may have, however, prevented suicides via increased adequacy of treatment, likely reflecting enhanced compliance.

We found no effect of antidepressant sales on female suicide, although the prevalence of women using antidepressants has almost tripled and the per-capita sales have more than quadrupled during our study period. Female suicides are uncommon and confidence intervals thus wide, so firm conclusions are difficult to draw. However, mere lack of statistical power is unlikely to explain our null finding since also the effect sizes among women were much smaller or even reversed compared to those among men. A Norwegian study from 1980–2004 found a beneficial effect of regional antidepressant sales on suicide rates only during low sales levels, and no effect during high sales [2]. This could also be true for Finnish women. The start of the decline in suicide rates in the early 1990s could have been caused by an increase in the then low levels of antidepressant sales whereas during the time of our study (1995–2007) further increase in antidepressant sales was no longer reducing female suicides.

Among men, the beneficial effect of antidepressant sales was only observed in some social groups, possibly reflecting differential likelihood of receiving and complying with treatment. Men with higher education, higher income, and employment may have benefited more of the increase in antidepressant prescribing if they have become more likely to be adequately treated when depressed. One reason behind this could relate to better detection of depression in higher social groups, although in Finland, detection of psychiatric disorders among patients presenting in primary care seems to be more likely among people with *low* education and social status, partly because of case severity [32]. People from higher social groups may also be more active in seeking treatment and adhere to medication [33], possibly due to more economic resources and better access to occupational health services. The seemingly contrasting result of a beneficial effect among those not living in owned housing and those living without a partner may reflect the fact that these groups also include all individuals living in institutional settings. The institutionalized group may have benefitted from increased antidepressant prescribing since due to their institutional residence their mental health problems are likely to be better detected and managed. However, since our data lack any information on antidepressants administered in institutional settings, this question is beyond the scope of our study. Men not living in owned housing and men living without a partner are high-risk groups for suicide and thus a beneficial effect observed in these groups may also reflect a success in targeting treatment.

The reasons behind alcohol-related suicide risk being unaffected by antidepressant sales require further investigation. Alcohol-related suicides may differ clinically from non-alcohol-related suicides. A Canadian psychological autopsy study found completers of alcohol-related suicides to be more impulsive and more aggressive, to have lower education and to have more psychiatric comorbidities than completers of non-alcohol-related suicides [34]. In Finland suicide attempters with alcohol-use disorders are less likely to receive and comply with appropriate aftercare [35], [36]. Correspondingly, in our data, only 10% of alcohol-related suicides were preceded by minimally adequate antidepressant treatment during the year of suicide, whereas for non-alcohol-related suicides the proportion was over 20%. Further studies are needed to assess whether this difference is due to differences in receipt of antidepressant treatment, compliance with it, or both. The alcohol-related suicide rate among Finnish men has stagnated in the 2000s at around 10 per 100,000 person years, which is very high compared with a 26-country-average *overall* suicide rate of 9.6 [16]. Although even in Finland, the bulk of suicides is non-alcohol-related and has reacted to the increase in antidepressant prescribing, the over 30% of male suicides that are alcohol-related have not, and call for more rigorous prevention strategies.

### Strengths and limitations

We used individual-level register data with nearly one million individuals and negligible loss to follow-up. The accuracy and reliability of the Finnish cause-of-death registration has been ranked among the best in international comparisons [37]. Particularly, as medico-legal autopsies are carried out in over 90% of accidental and violent deaths in people under 65 [38], alcohol intoxication as a contributory cause of suicide can be reliably assessed in our data.

Although we use individual-level data, our explanatory variable is measured at the regional level, not permitting us to assess the effect of individual-level antidepressant use on suicide risk. Our conclusions thus remain at the aggregate level: the causal connection between falling suicide rates and increasing antidepressant sales seems to be limited to non-alcohol-related male suicide.

We used region and time fixed-effects models to control for regional differences and time trends that may influence suicide risk irrespective of antidepressant sales. Previous aggregate-level studies reporting a significantly beneficial effect of antidepressant sales on suicide rates mostly use less stringent controls for time [1], [11], [13], [14], [17], with the exceptions of one Norwegian [2] and one cross-national study [16]. Controlling for time is essential for inferring causality between two co-occurring trends because it removes the confounding effects of all other co-occurring trends in observed or unobserved factors, and thus effects are only inferred from the variation between regions in the changes in antidepressant sales.

It should be noted, though, that adding the year fixed effects could be an over-adjustment if the year-to-year differences in suicide rates common to all regions were in fact brought about by the increase in overall antidepressant sales. In other words, if regional variation in trends is small then controlling for everything that is common across regions could mask beneficial effects of the sales increase. Crude regional correlations between antidepressant sales and suicide rates, however, implied that antidepressant sales could explain a maximum of 40% of non-alcohol-related male suicide rate, but less than 10% of alcohol-related male suicide rate and 0–10% of female suicide rate. We thus believe that if there has been a causal effect of increased antidepressant sales on suicide rates it has mainly occurred for non-alcohol-related male suicides.

One limitation to our study is that regional-level antidepressant data was only available from 1995 onwards and thus excludes the period from the end of the 1980s when non-tricyclic antidepressants were first introduced and the decline in suicide rates began in Finland [3]. In particular since previous studies have shown stronger effects during times of lower antidepressant sales [2], [17], our results are likely to underestimate any effect that the introduction and early expansion of non-tricyclic antidepressant before 1995 may have had on the declining suicide rates. However, the decline in suicide rates has continued throughout our study period and thus we should be able to observe effects of increasing antidepressant sales.

Our results were mostly robust to different model specifications and different measurement of antidepressant sales. However, the significant reduction in non-alcohol-related male suicide with increased proportion of antidepressant users receiving minimally adequate treatment was not replicated when controlling for time as a national linear trend or as regional linear trends, when assessing suicide risk with one-year lag with respect to antidepressant sales, or when minimally adequate treatment was defined as a minimum of 180 defined daily doses instead of 90. We thus remain cautious with the interpretation of a beneficial effect of rising antidepressant sales on suicide risk among men.

### Conclusions

We used individual-level register-data to study the association between regional antidepressant sales and the risk for non-alcohol-related and alcohol-related suicide in Finland in 1995–2007. Increased proportion of antidepressant users who received doses reflecting minimally adequate treatment may have prevented non-alcohol-related suicides among men.

## Supporting Information

Table S1 **Age-adjusted relative risk (RR) of suicide and 95% confidence interval (CI) per unit change in regional non-tricyclic antidepressant sales from sensitivity analyses using different model specifications and antidepressant definitions.**
(DOC)
