# Supplementary material for: Antidepressant Sales and the Risk for Alcohol-Related and Non-Alcohol-Related Suicide in Finland—An Individual-Level Population Study
Source: PLoS One. 2014 Jun 3;9(6):e98405. doi: 10.1371/journal.pone.0098405 (PMC4043885; doi:10.1371/journal.pone.0098405)
Supplement: Table S1 — Age-adjusted relative risk (RR) of suicide and 95% confidence interval (CI) per unit change in regional non-tricyclic antidepressant sales from sensitivity analyses using different model specifications and antidepressant definitions. (DOC) [file pone.0098405.s001.doc]

| **Table S1. Age-adjusted relative risk (RR) of suicide and 95% confidence interval (CI) per unit change in regional non-tricyclic antidepressant sales from sensitivity analyses using different model specifications and antidepressant definitions** | | | | | | | | | | | | | | | | | |
| --- | --- | --- | --- | --- | --- | --- | --- | --- | --- | --- | --- | --- | --- | --- | --- | --- | --- |
|  |  | | 1 | | | 2 | | 3 | | 4 | | 5 | | 6 | | 7 | |
| Men | | | RR | | 95% CI | RR | 95% CI | RR | 95% CI | RR | 95% CI | RR | 95% CI | RR | 95% CI | RR | 95% CI |
| All suicides | | |  | |  |  |  |  |  |  |  |  |  |  |  |  |  |
|  | Sold doses^1^ | | 1.005 | | 0.971-1.041 | 1.005 | 0.970-1.043 | 1.007 | 0.976-1.039 | 1.026 | 0.982-1.072 | 1.018 | 0.982-1.056 | 1.011 | 0.979-1.045 |  |  |
|  | Prevalence^2^ | | 1.020 | | 0.904-1.150 | 1.020 | 0.936-1.111 | 1.037 | 0.930-1.156 | 1.018 | 0.890-1.163 | 1.040 | 0.922-1.174 | 1.031 | 0.927-1.146 |  |  |
|  | % min adeq^3^ | | 0.993 | | 0.984-1.003 | 0.993 | 0.981-1.005 | 0.995 | 0.988-1.002 | 0.997 | 0.990-1.005 | 0.999 | 0.989-1.009 | 0.993 | 0.982-1.003 | 1.006 | 0.997-1.015 |
| Non-alcohol-related | | | | | | | | | | | | | | | | | |
|  | Sold doses | 1.000 | | | 0.959-1.042 | 1.000 | 0.969-1.032 | 0.998 | 0.962-1.037 | 1.015 | 0.964-1.069 | 1.003 | 0.960-1.049 | 1.007 | 0.968-1.046 |  |  |
|  | Prevalence | 1.034 | | | 0.895-1.195 | 1.034 | 0.945-1.132 | 1.066 | 0.936-1.215 | 1.077 | 0.918-1.264 | 0.975 | 0.842-1.129 | 1.052 | 0.927-1.193 |  |  |
|  | % min adeq | 0.987 | | | 0.976-0.998 | 0.987 | 0.977-0.998 | 0.996 | 0.987-1.004 | 0.999 | 0.990-1.008 | 0.999 | 0.987-1.011 | 0.984 | 0.971-0.997 | 1.001 | 0.990-1.012 |
| Alcohol-related | | | | | | | | | | | | | | | | | |
|  | Sold doses | 1.019 | | | 0.958-1.083 | 1.019 | 0.945-1.098 | 1.027 | 0.969-1.088 | 1.040 | 0.959-1.129 | 1.050 | 0.986-1.119 | 1.024 | 0.966-1.085 |  |  |
|  | Prevalence | 0.999 | | | 0.805-1.238 | 0.999 | 0.778-1.282 | 0.974 | 0.801-1.184 | 0.881 | 0.690-1.123 | 1.204 | 0.975-1.486 | 0.993 | 0.819-1.203 |  |  |
|  | % min adeq | 1.006 | | | 0.989-1.023 | 1.006 | 0.986-1.026 | 0.994 | 0.981-1.007 | 0.996 | 0.982-1.010 | 0.998 | 0.980-1.017 | 1.011 | 0.992-1.032 | 1.016 | 0.999-1.033 |
| Women | |  | | |  |  |  |  |  |  |  |  |  |  |  |  |  |
| All suicides | |  | | |  |  |  |  |  |  |  |  |  |  |  |  |  |
|  | Sold doses | 1.005 | | | 0.960-1.053 | 1.005 | 0.956-1.056 | 1.001 | 0.961-1.043 | 0.986 | 0.928-1.046 | 1.025 | 0.976-1.077 | 1.004 | 0.961-1.049 |  |  |
|  | Prevalence | 1.025 | | | 0.860-1.222 | 1.025 | 0.845-1.244 | 1.012 | 0.878-1.166 | 0.991 | 0.838-1.173 | 1.069 | 0.902-1.266 | 1.065 | 0.909-1.247 |  |  |
|  | % min adeq | 1.000 | | | 0.976-1.024 | 1.000 | 0.971-1.029 | 0.997 | 0.983-1.010 | 0.995 | 0.980-1.009 | 0.995 | 0.971-1.020 | 0.997 | 0.967-1.027 | 0.993 | 0.971-1.014 |
| Non-alcohol-related | | | | | | | | | | | | | | | | | |
|  | Sold doses | 0.997 | | | 0.949-1.048 | 0.997 | 0.942-1.055 | 0.994 | 0.952-1.038 | 0.982 | 0.922-1.045 | 1.022 | 0.970-1.076 | 0.995 | 0.949-1.042 |  |  |
|  | Prevalence | 1.021 | | | 0.846-1.231 | 1.021 | 0.838-1.243 | 1.016 | 0.873-1.181 | 0.993 | 0.831-1.187 | 1.075 | 0.899-1.286 | 1.028 | 0.868-1.217 |  |  |
|  | % min adeq | 0.997 | | | 0.972-1.022 | 0.997 | 0.967-1.027 | 0.999 | 0.984-1.014 | 0.998 | 0.983-1.014 | 0.987 | 0.961-1.013 | 0.994 | 0.963-1.027 | 0.993 | 0.971-1.016 |
| Alcohol-related | | | | | | | | | | | | | | | | | |
|  | Sold doses | 1.060 | | | 0.935-1.202 | 1.060 | 0.938-1.197 | 1.053 | 0.940-1.179 | 1.004 | 0.821-1.226 | 1.052 | 0.919-1.203 | 1.072 | 0.953-1.207 |  |  |
|  | Prevalence | 1.053 | | | 0.642-1.725 | 1.052 | 0.642-1.726 | 0.980 | 0.645-1.487 | 1.065 | 0.638-1.776 | 1.027 | 0.618-1.708 | 1.366 | 0.877-2.127 |  |  |
|  | % min adeq | 1.025 | | | 0.956-1.098 | 1.025 | 0.945-1.111 | 0.979 | 0.941-1.018 | 0.976 | 0.935-1.018 | 1.071 | 0.992-1.156 | 1.016 | 0.928-1.113 | 0.993 | 0.933-1.058 |
| Region fixed effects | | | | yes | | yes | | yes | | yes | | yes | | yes | | yes | |
| Time fixed effects | | | | yes | | yes | | yes | | yes | | yes | | yes | | yes | |
| **Sensitivity analyses**: 1: Logistic regression; 2: Standard errors clustered on the regional level; 3: National linear time trend; 4: Regional linear time trends; 5: Suicide assessed with one-year lag with respect to antidepressant sales; 6: Tricyclic antidepressants not excluded; 7: Minimally adequate doses defined as at least 180 DDDs  ^1^Sold doses per capita; ^2^Prevalence of antidepressant users; ^3^Proportion of antidepressant users with yearly purchases reflecting minimally adequate treatment | | | | | | | | | | | | | | | | | |
